# propr: An R-package for Identifying Proportionally Abundant Features Using Compositional Data Analysis

**DOI:** 10.1038/s41598-017-16520-0

**Published:** 2017-11-24

**Authors:** Thomas P. Quinn, Mark F. Richardson, David Lovell, Tamsyn M. Crowley

**Affiliations:** 1 Deakin University, Bioinformatics Core Research Group, Geelong, Victoria, Australia; 2 Deakin University, Centre for Molecular and Medical Research, Geelong, Victoria, Australia; 3 Deakin University, Centre for Integrative Ecology, Geelong, Victoria, Australia; 4 Queensland University of Technology, Brisbane, Queensland Australia

## Abstract

In the life sciences, many assays measure only the relative abundances of components in each sample. Such data, called *compositional* data, require special treatment to avoid misleading conclusions. Awareness of the need for caution in analyzing compositional data is growing, including the understanding that correlation is not appropriate for relative data. Recently, researchers have proposed proportionality as a valid alternative to correlation for calculating pairwise association in relative data. Although the question of how to best measure proportionality remains open, we present here a computationally efficient R package that implements three measures of proportionality. In an effort to advance the understanding and application of proportionality analysis, we review the mathematics behind proportionality, demonstrate its application to genomic data, and discuss some ongoing challenges in the analysis of relative abundance data.

## Introduction

Advances in the technology used to assay biological systems have led to a rapid increase in the amount of data generated. However, there has not yet emerged a clear consensus over the best method for analyzing these data. In fact, some of the most commonly used methods fundamentally ignore the underlying nature of the data studied. Count data, such as those produced by high-throughput sequencing, belong within the domain of relative data. Other examples of relative data include data generated by RNA-sequencing (RNA-Seq), chromatin immunoprecipitation sequencing (ChIP-Seq), Methyl-Capture sequencing, 16S amplicon-sequencing, and metabolomics. Methods that ignore the relative nature of these data yield erroneous results.

Compositional data have two key geometric properties. First, the total sum of all component values is an artifact of the sampling procedure^1^. Second, the distance between component values is only meaningful proportionally (e.g., the difference between 100 and 200 counts carries the same information as the difference between 1000 and 2000 counts)^1^. Biological count data (e.g., gene expression data from RNA-Seq), as relative data, also have these properties. Any number of factors, such as technical variability or differences in experiment-specific abundance, can impact the total number of counts (sometimes called the library size). Simply dividing count data by the library size does not address the systematic biases in RNA-Seq data. Although several normalization strategies exist, the choice of method can drastically change the number and identity of genes reported as differentially expressed^2^. This sensitivity to normalization holds true for other modes of compositional data as well^3^. Moreover, some methods, such as trimmed mean of M (TMM) normalization, give different results depending on how lowly expressed genes get removed from the data^2^.

By ignoring the relative nature of biological count data, investigators implicitly assume that absolute differences between counts have meaning^4^. That is, they assume the data exist in real Euclidean space^1^. This may explain why there has emerged a number of hyper-parameterized, assay-specific normalization methods used in the calculation of differential abundance that fail to generalize to data produced by other assays^4^. Meanwhile, methods that accommodate compositional data, such as the ALDEx2 package for R, aim to offer a unified way to compute differential abundance regardless of the data source^4^. However, the ALDEx2 package lacks a way to measure association in relative data.

Correlation has often been applied inappropriately to compositional data in the life sciences^5^. As Pearson warned in 1896, correlation gives spurious results when applied to relative data: i.e., given three statistically independent variables, X, Y, and Z, the ratios X/Z and Y/Z will correlate with one another by virtue of their shared denominator^6^. If we consider that Z may represent, for example, the library size, we see how two uncorrelated features, X and Y, may appear correlated even when they are not. Spurious correlation is not merely a statistical concern: when applied to real biological data, correlation can lead to wrong conclusions^5,7^. For example, one might incorrectly conclude that there exists a coordinated regulation among a module of transcriptionally independent genes.

As an alternative to correlation, proportionality is a measure of association that is valid for compositional data^5,8^. Borrowing from compositional data analysis (CoDA) principals, this approach uses a log-ratio transformation (*LR) of the original feature vectors in order to transpose the data from a simplex into real Euclidean space^1,9^. These transformed abundances then provide a substrate for calculating the *log-ratio variance* (VLR), defined as the variance of the ratio of two log transformed feature vectors (var*LR(*X*)/*LR(*Y*))^9^. Interestingly, the VLR is the same for relative values and their absolute equivalent. However, the VLR lacks a scale that would otherwise make it possible to compare dependency across multiple feature pairs. In essence, what we call proportionality is a modification to the VLR that establishes scale.

The propr package, now available through the Comprehensive R Archive Network (CRAN), implements two measures of proportionality, *ϕ*^5^ and *ρ*_*p*_^8^ (defined formally in the next section). Like the Pearson correlation coefficient, the metric *ρ*_*p*_ is naturally symmetric in its arguments. However, we can make *ϕ* naturally symmetric too by slightly altering its definition, as shown in the next section. Otherwise, *ρ*_*p*_ ranges from [−1, 1], reinforcing its analogy to correlation, while *ϕ* ranges from [0, ∞), reinforcing its analogy to dissimilarity. Yet, it is the conceptual and mathematical link between *ϕ* and *ρ*_*p*_ metrics that allows us to present the propr package as a single portal to proportionality analysis.

## Methods

Consider a matrix of *D* features (as columns) measured across *N* samples (as rows) exposed to a binary or continuous event. This event might involve case-control status, treatment status, treatment dose, or time. Proportionality, as analogous to, but distinct from, correlation, measures the association between two log-ratio transformed feature vectors. By default, this package uses the centered log-ratio transformation (clr); this transformation scales each subject vector by its geometric mean (indicated as *g*(*x*)). However, we also include an implementation of the additive log-ratio transformation (alr). These transformations get applied to each subject vector, *x*, according to the following definitions:

1 $${\rm{clr}}(x)=[\mathrm{ln}\,\frac{{x}_{i}}{g(x)};\mathrm{...};\,\mathrm{ln}\,\frac{{x}_{D}}{g(x)}]$$

2 $${\rm{alr}}(x)=[\mathrm{ln}\,\frac{{x}_{i}}{{x}_{D}};\mathrm{...;}\,\mathrm{ln}\,\frac{{x}_{D-1}}{{x}_{D}}]$$

Applying a log-ratio transformation to each sample results in a new matrix, *A*, containing *N* rows (as samples) and *D* columns (as features). The propr package implements three measures of proportionality in the R language, as defined for *A*. Note that although we calculated each row of *A* using Eq. 1 or Eq. 2, we use the columns of this matrix to calculate proportionality. Two principal functions, phit and perb, calculate the proportionality metrics $\varphi$ (Eq. 3)^5^ and *ρ*_*p*_ (Eq. 4)^8^, respectively. The functions phit and perb return a matrix of *D*_2_ elements relating each combination of log-ratio transformed feature vectors, *A*_*i*_ and *A*_*j*_ (*i*, *j* ∈ *D*), according to the following definitions:

3 $$\varphi ({A}_{i},{A}_{j})=\frac{var({A}_{i}-{A}_{j})}{var({A}_{i})}$$

4 $${\rho }_{p}({A}_{i},{A}_{j})=1-\frac{var({A}_{i}-{A}_{j})}{var({A}_{i})+var({A}_{j})}$$

In addition, we provide the phis function to calculate *ϕ*_*s*_ (Eq. 5), a naturally symmetric variant of *ϕ*. Interestingly, *ϕ*_*s*_ relates to *ρ*_*p*_ by a monotonic function^8^, meaning you can calculate one from the other. This measure of proportionality relates the variance of the log-ratio (VLR) to the variance of the log-product (VLP), according to the following definition:

5 $${\varphi }_{s}({A}_{i},{A}_{j})=\frac{var({A}_{i}-{A}_{j})}{var({A}_{i}+{A}_{j})}$$

We refer the reader to the supplemental appendix for a demonstration of how these measures of proportionality relate to the slope, *β*, and the Pearson correlation coefficient, *r*, of pairwise log-ratio transformed data (S1 Appendix). This appendix also illustrates how *ϕ*, *ϕ*_*s*_, and *ρ*_*p*_ relate to one another.

In considering the two log-ratio transformations, alr uses a specified feature to transform the original subject vectors. When used in conjunction with an *a priori* known unchanged reference, alr effectively back-calculates the absolute counts from the relative components. By specifying, for example, a house-keeping gene or an experimentally fixed variable, the investigator can achieve a more accurate measure of dependence than through clr^8^. The user can toggle alr transformation in lieu of the default clr transformation by supplying the name of the unchanged reference (or references) to the ivar argument of the phit, perb, or phis function. In either case, we wish to alert the reader that log-ratio transformation, by its definition, require non-zero elements in the data matrix. As such, any log-ratio analysis must first address zeros. Yet, how best to do this remains an open question and a topic of active research^10^. For simplicity, propr automatically replaces all zero values with 1 prior to log-ratio transformation, corresponding to the multiplicative replacement strategy^10^.

The R language, despite its widespread popularity, suffers from poor performance when scaling to big data. Its “copy-on-modify” behavior, whereby each modification of an object creates a duplication of that object, along with slow for-loops, makes it an imperfect choice for computationally expensive tasks such as the one here. Therefore, in order to speed up the run time and reduce RAM overhead, we harness the Rcpp package to draft the computationally expensive portions of this tool in C++^11^.

This package also offers a number of wrapper functions to visualize proportionality when working with high-dimensional data. We provide extensive documentation for these plotting methods in the package vignette, “Understanding RNA-Seq Data through Proportionality Analysis”, hosted with the package on CRAN. Among these tools are those used to generate the figures included in the next section.

## Results

### Application of proportionality

As a use case, we re-analyze the raw RNA-Seq counts from an already published study on cane toad (*Rhinella marina*) evolution and adaptation^12^. Sugar cane farmers introduced cane toads to Australia in 1935 as a cane beetle pest control measure, but these toads quickly became invasive. This event now serves as a notable example of failed biological control. Initially introduced into northeastern Australia (Queensland, QLD), cane toads have since spread westwards across the continent to Western Australia (WA)^12^. This dataset contains muscle tissue RNA transcript counts for 20 toads sampled from two regions (10 per region) in the wild. The two regions sampled, which we will treat as the experimental groups, include the long colonized site of introduction in QLD and the front of the range expansion in WA^12^. In this analysis, we want to understand the differences in gene expression between the established and expanding populations. By demonstrating propr on public data, we provide a reproducible example of how proportionality analysis can converge on an established biological narrative. The reader can find these data bundled with the release of the package on CRAN.

**Figure 1:**
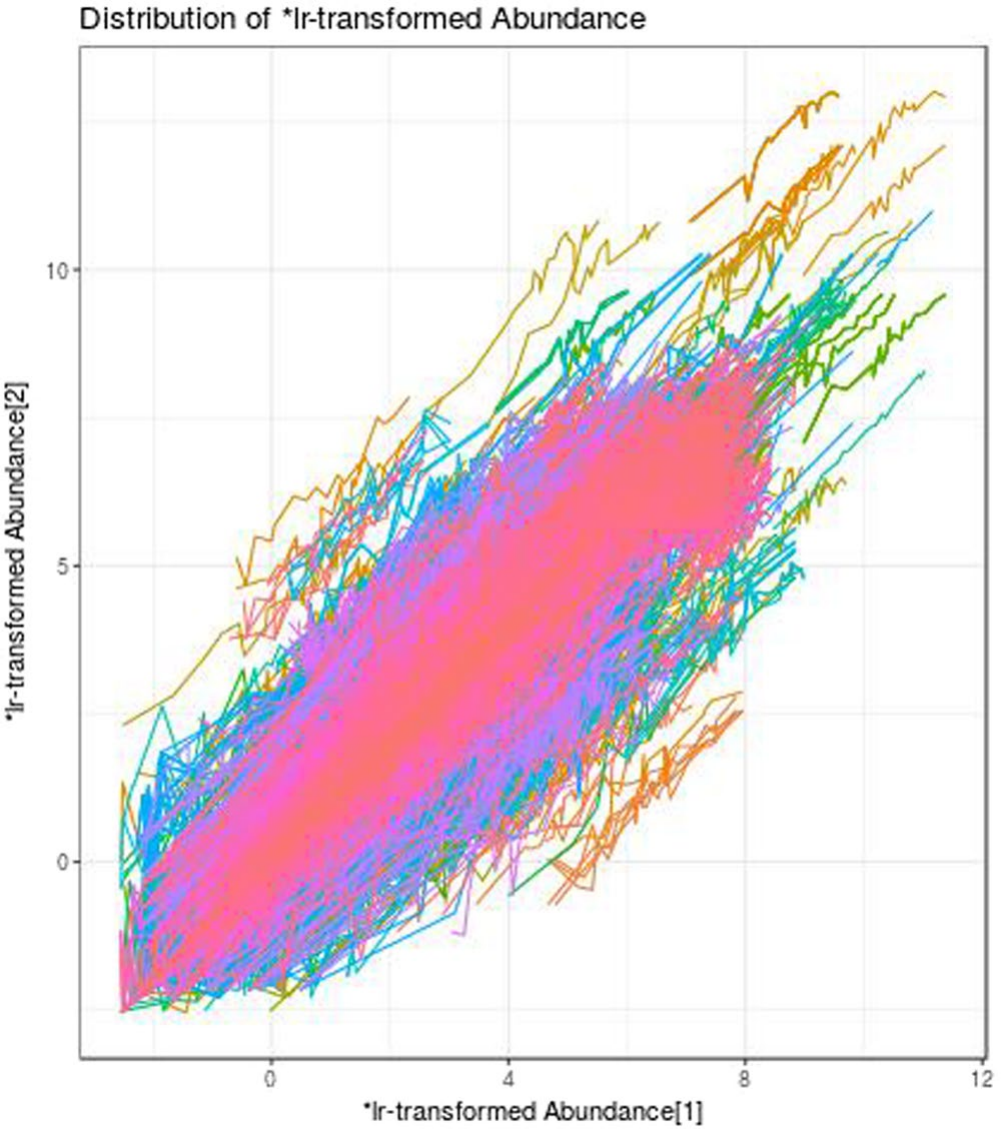
Smear plot. This figure shows the log-ratio abundance for each feature belonging to pairs index as highly proportional (*ρ*_*p*_ > 0.95). A smear of straight diagonal lines confirms that the feature pairs indexed as proportional actually show proportional abundance. In other words, large deviations from *y* = *x* indicate small values of |*ρ*_*p*_|. Figure produced using the smear function in propr.

We begin by constructing the proportionality matrix using all 57,580 transcript counts, yielding an *N*^2^ matrix 24.7 Gb in size. To minimize the number of lowly expressed transcripts included in the final result, we subset the matrix to include only those transcripts with at least 10 counts in at least 10 samples. By removing the features at this stage, we can exploit a computational trick to calculate proportionality and filter simultaneously, reducing the required RAM to only 5 Gb without altering the resultant matrix. Next, in the absence of a hypothesis testing framework, we arbitrarily select those “highly proportional” transcripts with *ρ*_*p*_ > 0.95. We refer the reader to the supplementary vignette for a justification of this cutoff (S1 Appendix). When plotting the pairwise log-ratio transformed abundances for these “highly proportional” transcript pairs, a smear of straight diagonal lines confirms that the feature pairs indexed as proportional actually show proportional abundance (Fig. 1).

**Figure 2:**
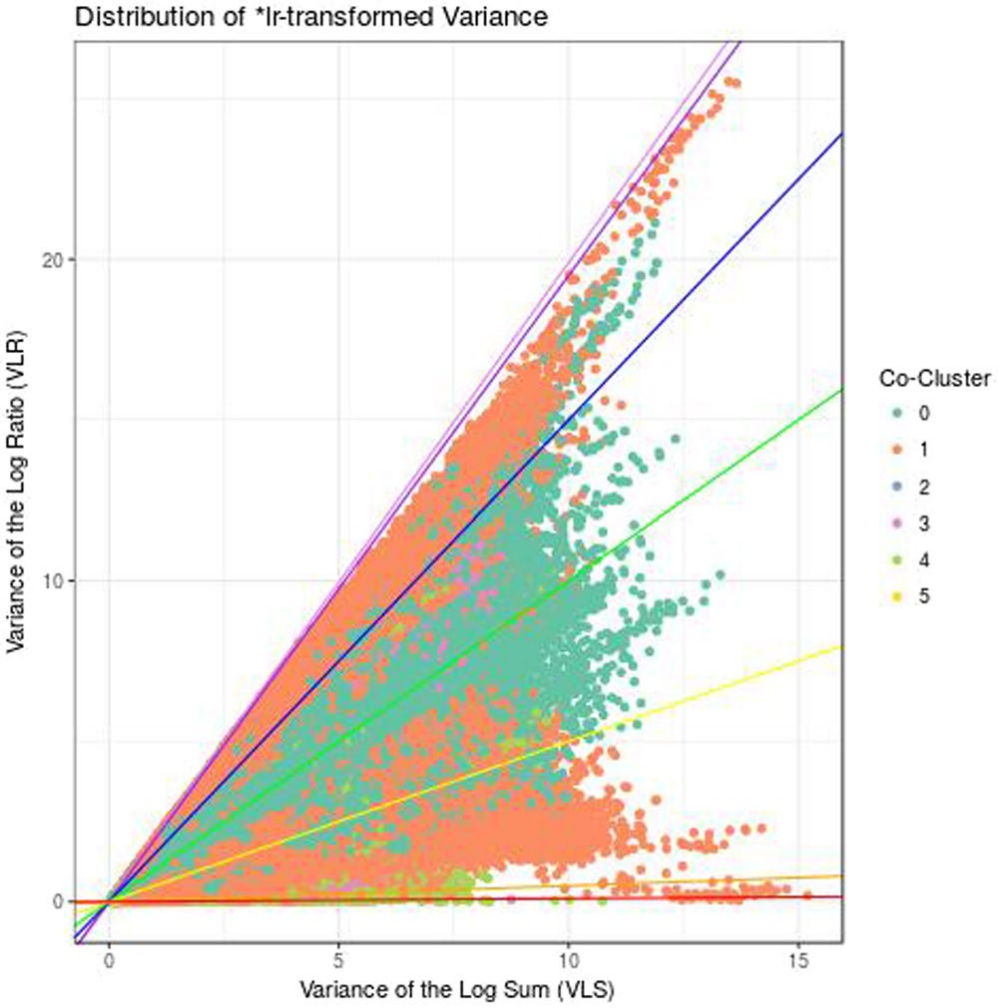
Prism plot. This figure shows the distribution of feature pairs according to the variance of the log-ratio (VLR) and the variance of the log sums (VLS) for all pairs in which at least one of the features participates in at least one highly proportional (*ρ*_*p*_ > 0.95) pair. If both features in a pair belong to the same cluster, they receive a non-zero color code. Clusters created hierarchically based on the matrix $1-|{\rho }_{p}|$. Figure produced using the prism function in propr.

The procedure used to parse through the proportionality matrix now depends on the experimental question. Here, we wish to identify a highly proportional transcript module that happens to show differential abundance across the experimental groups. In this example, we take an unsupervised approach by hierarchically clustering the highly proportional feature pairs based on the matrix 1−*|ρ*_*p*_|. We note here that one could instead cluster using *ϕ*_*s*_ directly. When clustering, we call two features co-clustered if they belong to the cluster after cutting the dendrogram. Then, we project the pairs across two axes of variance, the *variance of the log-ratio* (VLR) and *variance of the log sums* (VLS) such that $\frac{VLR}{VLS}=1-{\rho }_{p}$. In this formula, we see that as VLR approaches 0, *ρ*_*p*_ approaches 1. Meanwhile, the VLS, the sum of the individual variances of two features in that pair, adjusts the rate of this limit. Since we would expect a differentially expressed module to have a low VLR and a high VLS, we prioritize pairs in co-cluster 1 for subsequent analysis (Fig. 2).

**Figure 3:**
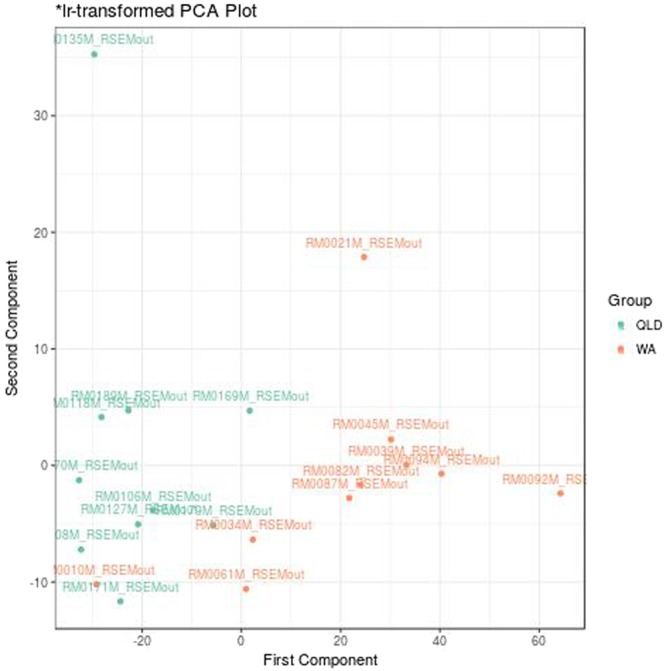
PCA plot. This figure shows all samples projected across the first two components of a principal components analysis (PCA), calculated using the log-ratio transformed data. This plot colors samples based on the experimental group. Figure produced using the pca function in propr.

Co-clusters containing feature pairs with a low VLR and a high VLS have the potential to explain differences between the experimental groups. However, the high VLS may not necessarily have anything to do with the experimental condition. For example, this co-cluster might instead include highly proportional features that show wide individual feature variance due to batch effects. For the cane toad data, however, the experimental condition does indeed seem to drive the high individual feature variances in the module, as evidenced by the near perfect group separation when visualizing the first two components of a principal components analysis (PCA) (Fig. 3). Note that this plot calculates PCA using the log-ratio transformed data, making it a statistically valid choice for compositional data^13^. The separation between groups achieved here compares to that reported in the original publication, which used features selected by the edgeR package^12^. In addition, gene set enrichment analysis of the gene ontology terms for co-cluster 1 (S1 Table) shows an enrichment for similar molecular functions as those enriched among the transcripts selected by edgeR (S2 Table), as well as those highlighted in the original publication^12^. This is particularly impressive considering that we have no reason to expect most differentially expressed transcripts would appear in differentially expressed modules too.

**Figure 4:**
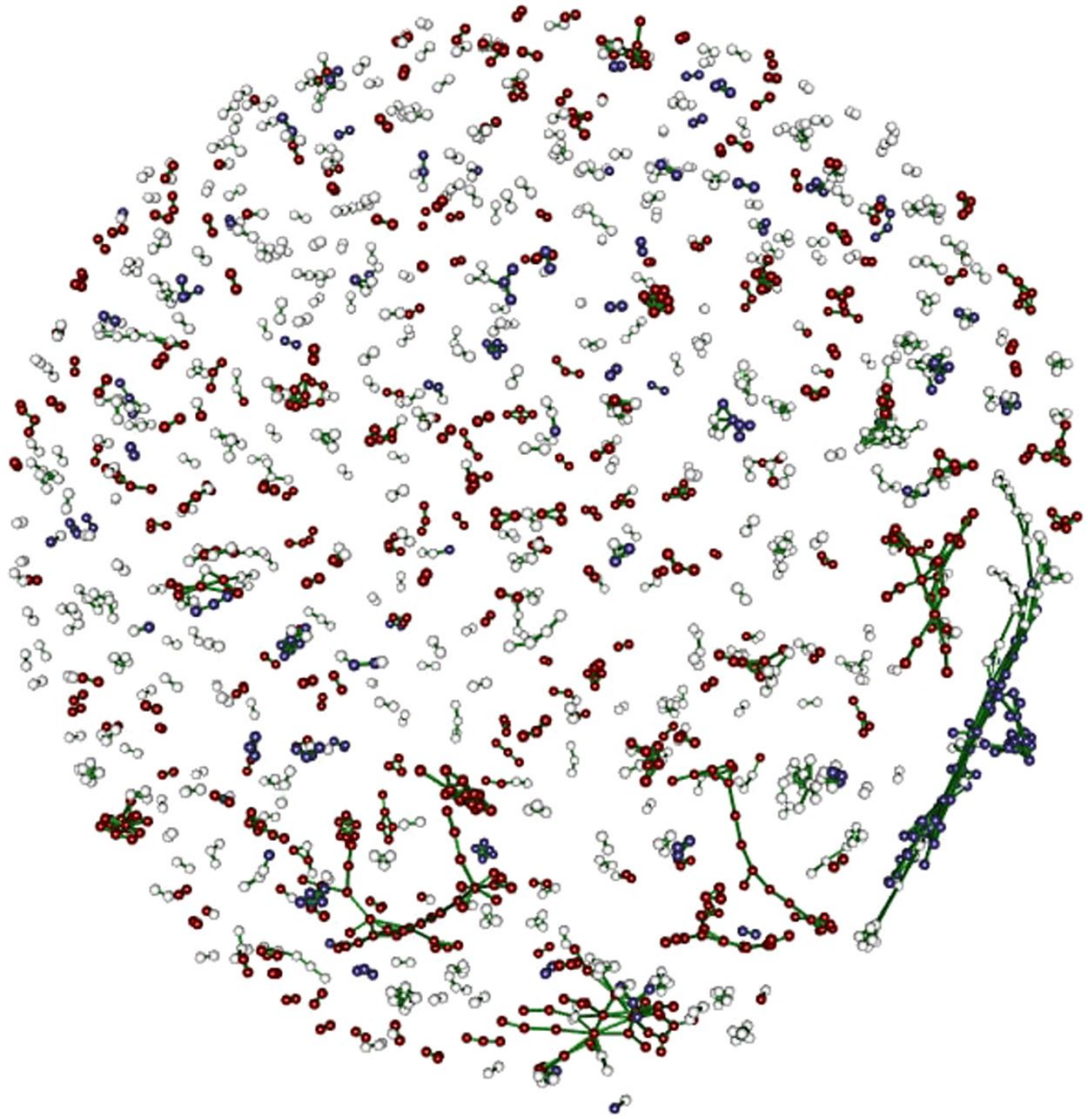
Network plot. This figure shows a 3D projection of all feature pairs indexed as highly proportional (*ρ*_*p*_ > 0.95) within co-cluster 1. Red nodes indicate transcripts with increased expression according to edgeR. Blue nodes indicate transcripts with decreased expression according to edgeR. White nodes indicate transcripts included in co-cluster 1, but not selected by edgeR. Importantly, we see here several highly proportional up-regulated and down-regulated modules. Figure produced using the cytescape function in propr.

Although the exact gene ontology terms differ by method, our approach yields a constellation of macromolecular metabolic terms that suggests a common story: “metabolic enzymes are overwhelmingly upregulated at the invasion front”^12^, interpreted this to mean that WA cane toads may experience more environmental stress than those from QLD^12^. Yet, while agreeing here, proportionality analysis offers an additional benefit in that it provides a layer of information on pairwise associations, all without requiring any kind of normalization. Nevertheless, we believe there exists added value in integrating the results of propr and conventional differential expression analysis, best visualized as a network graph (Fig. 4).

### Evaluation of proportionality

Above, we show how we can use proportionality to understand RNA-Seq data. Here, we move on to evaluate how well each of the three measures of proportionality performs as compared to the Pearson’s correlation coefficient of the absolute data (referred to as *absolute correlation*). For this, we need a dataset for which we know already the absolute abundances exactly. Since this is unknown in the cane toad data, we use two other datasets: (a) a simulated dataset with 1,000 random features (i.e., following a negative binomial distribution) and an additional non-random feature, and (b) a time series of yeast mRNA abundance after removal of a key nutrient^14^. Knowing the absolute abundances, we can make a corresponding relative dataset by dividing the counts (as rows) by the per-sample sum (i.e., the library size). Since the library size changes non-randomly across samples, this operation constrains the data in a way that introduces spurious correlations.

**Figure 5:**
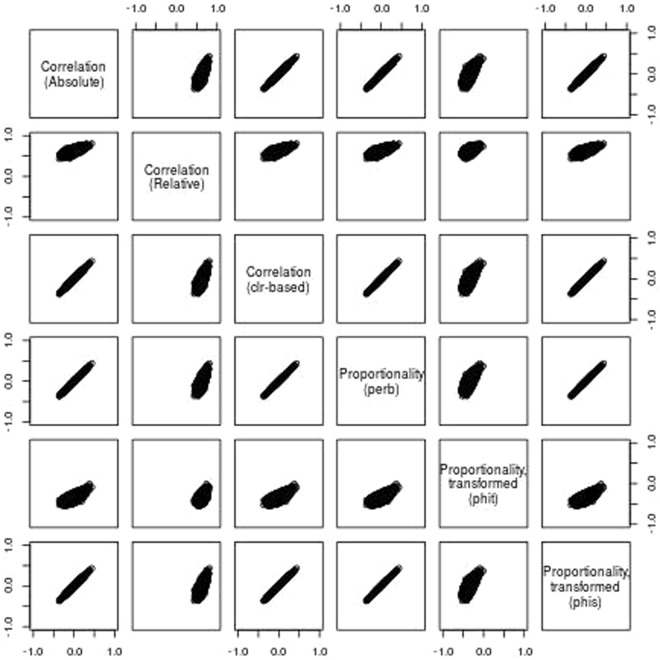
Evaluation of proportionality using simulated data. Using a simulated dataset with 1,000 random features and an additional non-random feature, this figure shows a scatter of 10,000 randomly sampled absolute correlations plotted against five other measures of dependence: (1) the correlation of relative data, (2) the correlation of clr-transformed data, (3) *ρ*_*p*_, (4) *ϕ*, and (5) *ϕ*_*s*_. To make the measures of *ϕ* and *ϕ*_*s*_ comparable, we transformed these from a range of [0, ∞) to [−1, 1]. From here, we can visually assess how well any two measures of dependence agree with one another.

For each dataset, we plot a scatter of 10,000 randomly sampled absolute correlations plotted against five other measures of dependence: (1) the correlation of relative data, (2) the correlation of clr-transformed data, (3) *ρ*_*p*_, (4) *ϕ*, and (5) *ϕ*_*s*_. Note that, since *ϕ* and *ϕ*_*s*_ range from [0. ∞), we have transformed these measures to make them comparable with correlation directly. For this, we use the function $f(x)=1-2\,{\rm{logistic}}(\mathrm{log}(x))$, such that ${\rho }_{p}=f({\varphi }_{s})$. Figure 5, built from the simulated data, shows the presence of many spurious correlations (Fig. 5: panel “Correlation (Absolute)” vs. “Correlation (Relative)”). Yet, these spurious correlations disappear when measuring proportionality (Fig. 5: panel “Correlation (Absolute)” vs. “Proportionality (perb)”). Notably, the spurious correlations from these data also disappear when measuring the correlation of clr-transformed data (Fig. 5: panel “Correlation (Absolute)” vs. “Correlation (clr-based)”).

**Figure 6:**
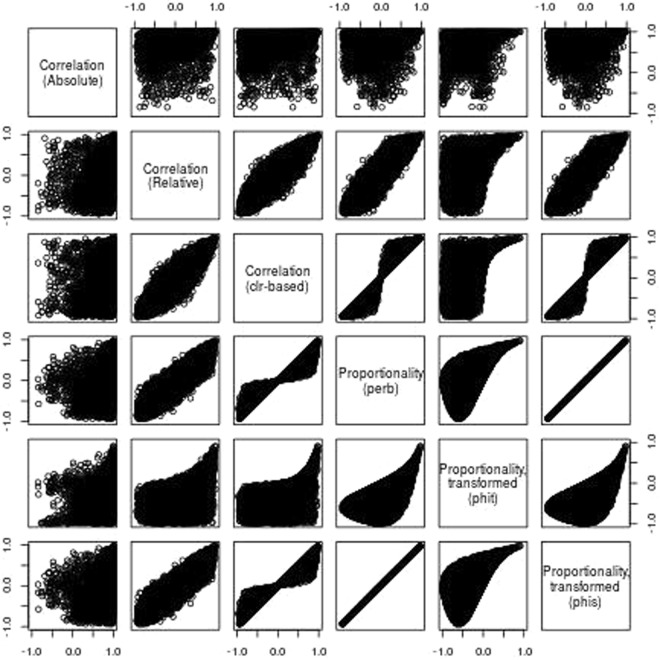
Evaluation of proportionality using yeast data. Using a time series of yeast mRNA abundance after removal of a key nutrient, this figure shows a scatter of 10,000 randomly sampled absolute correlations plotted against five other measures of dependence: (1) the correlation of relative data, (2) the correlation of clr-transformed data, (3) *ρ*_*p*_, (4) *ϕ*, and (5) *ϕ*_*s*_. To make the measures of *ϕ* and *ϕ*_*s*_ comparable, we transformed these from a range of [0, ∞) to [−1, 1]. From here, we can visually assess how well any two measures of dependence agree with one another.

Likewise, Fig. 6 uses the yeast data to extend an analysis provided in the supplemental materials of Lovell *et al*.^5^. As with the simulated data, we see here a number of spurious correlations (Fig. 6: panel “Correlation (Absolute)” vs. “Correlation (Relative)”). However, unlike with the simulated data, proportionality does not perfectly approximate the absolute correlation coefficients; still, proportionality does mitigate the presence and extent of the spurious events (as evidenced by the tapering of the scatter plot) (Fig. 6: panel “Correlation (Absolute)” vs. “Proportionality (perb)”). In other words, pairs that exhibit high proportionality (i.e., large *ρ*_*p*_ or, equivalently, small *ϕ* and *ϕ*_*s*_) also exhibit high absolute correlation, making proportionality a precise predictor of absolute correlation. Interestingly, unlike with the simulated data, clr-based correlation appears to produce more spurious events (Fig. 6: panel “Correlation (Absolute)” vs. “Correlation (clr-based)”) than proportionality, suggesting that proportionality may have some inherent advantage when applied to compositional data.

To quantify what we see visually, we compare each measure to the absolute correlation by tabulating the mean squared error (MSE), the number of spurious events, and the MSE of those spurious events (S3 Table). This confirms that, at least for the yeast data, proportionality results in fewer and less extreme spurious events. Note that, for both datasets, *ρ*_*p*_ and *ϕ*_*s*_ perform equally well because *ϕ*_*s*_ is a transformed variant of *ϕ*_*s*_^8^. Although *ϕ* produces even fewer spurious results than the other proportionality measures, this comes at the cost of greater total MSE. We refer the reader to the supplementary materials for a script that contains everything required to reproduce all analyses presented here.

## Discussion

The propr package for R, now available on CRAN, provides a fast implementation of three measures of proportionality, previously shown to rectify the issue of spurious correlation in the setting of compositional data^5,8^. These implementations offer a valid alternative to correlation that can accurately identify associated features in relative data^8^. By using the Rcpp package to draft the computationally expensive code in C++, the propr package achieves greater performance than possible in the native R environment without altering the front-end user experience^11^. In this way, proportionality analysis executes nearly as fast as base R correlation while retaining a simple programming interface. Yet, proportionality analysis is not without limitations.

First, unlike the *log-ratio variance* (VLR), the values of *ϕ* and *ρ*_*p*_ will change in the setting of missing feature data. Since biological assays rarely, if ever, capture all possible feature information, this property makes the centered log-ratio transformation (clr) a sub-optimal choice. The additive log-ratio transformation (alr), which allows the user to scale their data by a feature with an *a priori* known fixed abundance, such as a house-keeping gene or an experimentally fixed variable (e.g., a ThermoFisher ERCC synthetic RNA “spike-in”^15^), may provide a superior alternative. In contrast to clr, proportionality calculated with alr does not change with missing feature data because it effectively back-calculates the absolute feature abundance. Still, clr is probably sufficient when most features remain unchanged across samples, an assumption also built into the trimmed mean of M (TMM) normalization used in the analysis of RNA-Seq data^8,16^. Moreover, errors associated with clr transformation consist almost entirely of false negatives, rather than false positives, making it suitable for many scientific applications^8^.

Second, proportionality analysis, by nature of the log-ratio transformation, fails in the presence of zero values. As such, proportionality analysis inherits the zero replacement controversy prominent in the compositional data analysis literature. By default, propr replaces all zero values with 1. If analysts wish to explore other approaches to zero replacement–including conducting analyses of the sensitivity of results to zero imputation–they can so by manipulating the data prior to running routines in propr. We stress that, depending on the number of zero values in the data, replacement of zeros can have a major impact on the conclusions drawn from any log-ratio based analysis. Analysts should carefully examine their results to understand the extent to which they depend on the zero replacement strategy chosen.

Third, biological count data do not exist as true compositional data, but rather as a kind of “count-compositional” data, whereby small non-zero counts pose a unique challenge to analysis. This follows from how the log-ratio methods of compositional data analysis assume data to consist of *D* positive, real-valued components (i.e., a sample space of ${{\mathbb{R}}}_{+}^{D}$). Instead, count-compositional data consist of *D* non-negative, *integer-valued* components (i.e., a sample space of ${{\mathbb{Z}}}_{0+}^{D}$). The smaller the counts, the more noticeable the discretization of the count-compositional data becomes. Likewise, with smaller counts, the impact of sampling variation becomes more noticeable: additive variation affects the relative abundance of small counts more than large counts. Put succinctly, the difference between 1 count and 2 counts does not carry the exact same information as the difference between 1000 counts and 2000 counts. In practice, this means that a minor variation in small counts can have a major impact on the conclusions drawn from any log-ratio based analysis. Like with zero replacement, analysts should carefully examine their results to understand this sensitivity. We note that prior work has found substantial differences in how test statistics handle low-count genes in RNA-Seq^17^. For the purposes of demonstrating propr, we avoid this issue partly by removing from analysis any component with a predominance of low counts.

Finally, proportionality analysis currently lacks a hypothesis testing framework. Its distribution of heteroscedastic variance makes it unsuited for the z-transformation used to calculate the variance of the correlation statistic. Although more work is needed to create a rigorous framework for applying statistical tests to proportionality analysis, we include a supplementary vignette that offers a practical guide to choosing a proportionality cutoff (S1 Appendix). Nevertheless, we believe the propr package comes a long way in improving the accessibility of proportionality analysis to researchers. We hope many biological investigations can benefit from this alternative to correlation.

### Data availability

All data generated or analyzed during this study are included in this published article (and its Supplementary Information files).

## Electronic supplementary material

S1 Appendix

S1 Notes

S1 Data

S2 Data

S3 Data

S1 Table

S2 Table

S3 Table
