# Supplementary material for: propr: An R-package for Identifying Proportionally Abundant Features Using Compositional Data Analysis
Source: Sci Rep. 2017 Nov 24;7:16252. doi: 10.1038/s41598-017-16520-0 (PMC5701231; doi:10.1038/s41598-017-16520-0)
Supplement: Supplementary file 1 — S1 Appendix [file 41598_2017_16520_MOESM1_ESM.pdf]

propr: An R-package for Identifying Proportionally Abundant Features Using Compositional Data Analysis

Thomas P. Quinn, Mark F. Richardson, David Lovell, and Tamsyn M. Crowley

S1 Appendix: Examination of proportionality

Here, we set out the relationships between the slope (β) and r^2 values of pairwise log-transformed data, and the proportionality statistics ϕ, ϕ_s and ρ_p implemented in propr.

Lovell et al. proposed the "goodness-of-fit to proportionality" statistic ϕ to assess the extent to which a pair of random variables (x, y) are proportional [3]. This statistic was motivated by challenges in interpreting *logratio variance*, $\text{var}(\log(x/y))$, as a measure of association for variables that carry only relative information [1]. Logratio variance can be factored into two more interpretable terms:

$$\begin{aligned}
\text{var}(\log(x/y)) &= \text{var}(\log x - \log y) \\
&= \text{var}(\log x) + \text{var}(\log y) - 2\text{cov}(\log x, \log y) \\
&= \text{var}(\log x) \cdot \left(1 + \frac{\text{var}(\log y)}{\text{var}(\log x)} - 2\sqrt{\frac{\text{var}(\log y)}{\text{var}(\log x)}} \frac{\text{cov}(\log x, \log y)}{\sqrt{\text{var}(\log x)\text{var}(\log y)}}\right) \\
&= \text{var}(\log x) \cdot (1 + \beta^2 - 2\beta|r|) \\
&\triangleq \text{var}(\log x) \cdot \phi(\log x, \log y) \qquad (1)
\end{aligned}$$

where β is the *standardized major axis* estimate [4] of slope of random variables $\log y$ on $\log x$, and r the correlation between those variables. The first term in Equation 1, $\text{var}(\log x)$, is solely about the magnitude of variation at play and has nothing to do with y. The second term, ϕ, describes the degree of proportionality between x and y.

Lovell et al. [3] point out that $\phi(\log x, \log y)$ is not symmetric in its arguments. However, a symmetric alternative can be derived as follows. Using similar algebra to Equation 1, one can show that

$$\begin{aligned}
\text{var}(\log(x \cdot y)) &= \text{var}(\log x + \log y) \\
&= \text{var}(\log x) \cdot (1 + \beta^2 + 2\beta|r|)
\end{aligned}$$

and then define

$$\begin{aligned}
\phi_s(\log x, \log y) &\triangleq \frac{\text{var}(\log(x/y))}{\text{var}(\log(x \cdot y))} \\
&= \frac{\text{var}(\log x - \log y)}{\text{var}(\log x + \log y)} \\
&= \frac{1 + \beta^2 - 2\beta|r|}{1 + \beta^2 + 2\beta|r|}. \qquad (2)
\end{aligned}$$

Erb and Notredame refer to the square root of Equation 2 as $\tilde{\beta}$ in [2] and also show how this symmetric measure of proportionality relates to $\rho_p(\log x, \log y)$:

$$\begin{aligned}
\rho_p(\log x, \log y) &\triangleq \frac{2\text{cov}(\log x, \log y)}{\text{var}(\log x) + \text{var}(\log y)} \\
&= \frac{2\beta|r|}{1 + \beta^2} \\
&= \frac{1 - \phi_s(\log x, \log y)}{1 + \phi_s(\log x, \log y)}. \qquad (3)
\end{aligned}$$

References

[1] J Aitchison. *The Statistical Analysis of Compositional Data*. Chapman & Hall, Ltd., London, UK, UK, 1986.

[2] Ionas Erb and Cedric Notredame. How should we measure proportionality on relative gene expression data? *Theory in Biosciences = Theorie in Den Biowissenschaften*, 135(1-2):21–36, June 2016.

[3] David Lovell, Vera Pawlowsky-Glahn, Juan José Egozcue, Samuel Marguerat, and Jürg Bähler. Proportionality: a valid alternative to correlation for relative data. *PLoS computational biology*, 11(3):e1004075, March 2015.

[4] David I. Warton, Ian J. Wright, Daniel S. Falster, and Mark Westoby. Bivariate line-fitting methods for allometry. *Biological Reviews*, 81(2):259–291, May 2006.

How Proportional Is Proportional Enough?

Introduction

In this vignette, we use a real dataset to show how correlation analysis yields spurious results when applied to RNA-seq count data, and how proportionality analysis minimizes the discovery of falsely positive associations. We conclude by establishing a well-reasoned "rule of thumb" to answer the question, "How proportional is proportional enough?".

Correlation on relative data yields spurious results

Relative, or compositional, data do not exist in Euclidean space, but rather in a sub-space known as the Aitchison simplex (Aitchison 1986). As a consequence, calculating the Euclidean distance between points in relative space does not reflect the absolute distance between them. Many commonly used metrics implicitly assume that underlying data exist in Euclidean space; such metrics are invalid for relative data. Notably, this includes Pearson's correlation coefficient, a measure of the association between two variables (Lovell 2015).

What does it mean when we say that Pearson's correlation coefficient is invalid for relative data? When applied to relative data, including many of the biological datasets routinely studied, correlation yields erroneous results. Pearson first described this phenomenon in 1896 as spurious correlation (Pearson 1896). A spurious correlation is a falsely positive measure of association between two variables that are, in fact, not associated.

Below, we re-examine the dataset discussed by Lovell et al. (Lovell 2015) (as published by Marguerat et al. (Marguerat 2012)) which describes a time series of mRNA transcript abundance for yeast cells after the removal of a nutrient. The source data contain two parts: microarray data as expressed relative to the first time point, and a measure of the total RNA at timepoint 0. From these two parts, it is possible to approximate absolute RNA abundance at each timepoint.

Starting with propr version 2.2.0, the absolute abundances are available within the package itself for testing and exploration. The object, "marg.abs" corresponds to the object "Abs.t" as used in the supplementary materials of Lovell et al. (Lovell 2015). Note that we can make the absolute data relative by dividing each composition (i.e., time point) by the total sum of that composition. This constrains measurements to a proportion of the total, hence making them compositional.

```
library(propr)
data(marg.abs)
marg.rel <- t(apply(marg.abs, 1, function(x) x / sum(x)))
```

We now have two datasets: the first contains the absolute abundances of each transcript over time while the second contains the relative abundances of each transcript over time. Although this is microarray data, RNA-seq data is also compositional in that counts carry meaning only as a proportion of the total number of counts (i.e., library size). The compositional nature of RNA-seq data becomes more obvious when expressed, for example, as FPKM.

This dataset provides us with the opportunity to contrast the analysis of relative data with the analysis of the absolute counter-part. The importance of this evaluation comes from the reality that it is not always feasible to acquire data in absolute form. Note, however, that having access to absolute abundances usually makes the analysis of relative data superfluous. Below, we calculate Pearson's correlation coefficient for all pairs using both datasets.

```
Abs.cor <- stats::cor(marg.abs, use = "pairwise.complete.obs")
Rel.cor <- stats::cor(marg.rel, use = "pairwise.complete.obs")
```

Next, we plot a histogram of the absolute correlation coefficients. Interestingly, we see that this dataset contains a lot of truly (i.e., absolutely) positively correlated transcripts.

```r
plot(hist(Abs.cor))
```

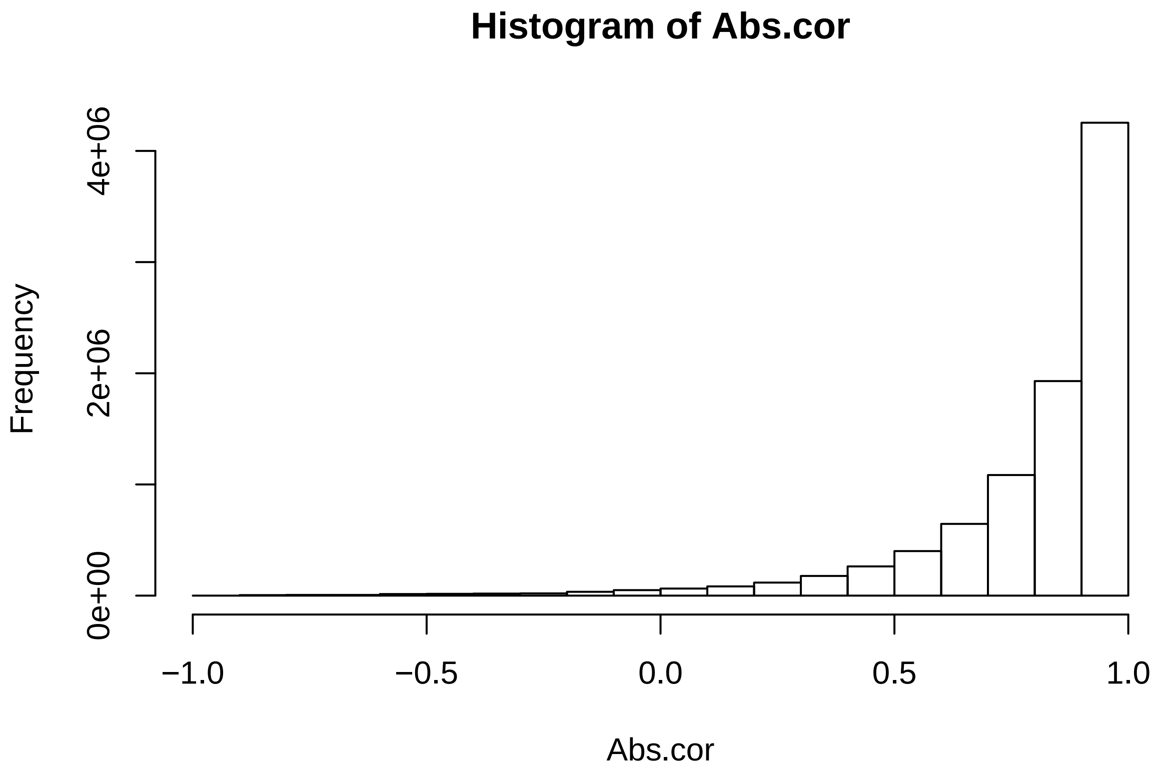

Perhaps surprisingly, there are fewer correlations when looking at the relative data than the absolute data. We emphasize here that *spurious correlations* does not mean *more correlations*, but rather *wrong correlations*.

```r
plot(hist(Rel.cor))
```

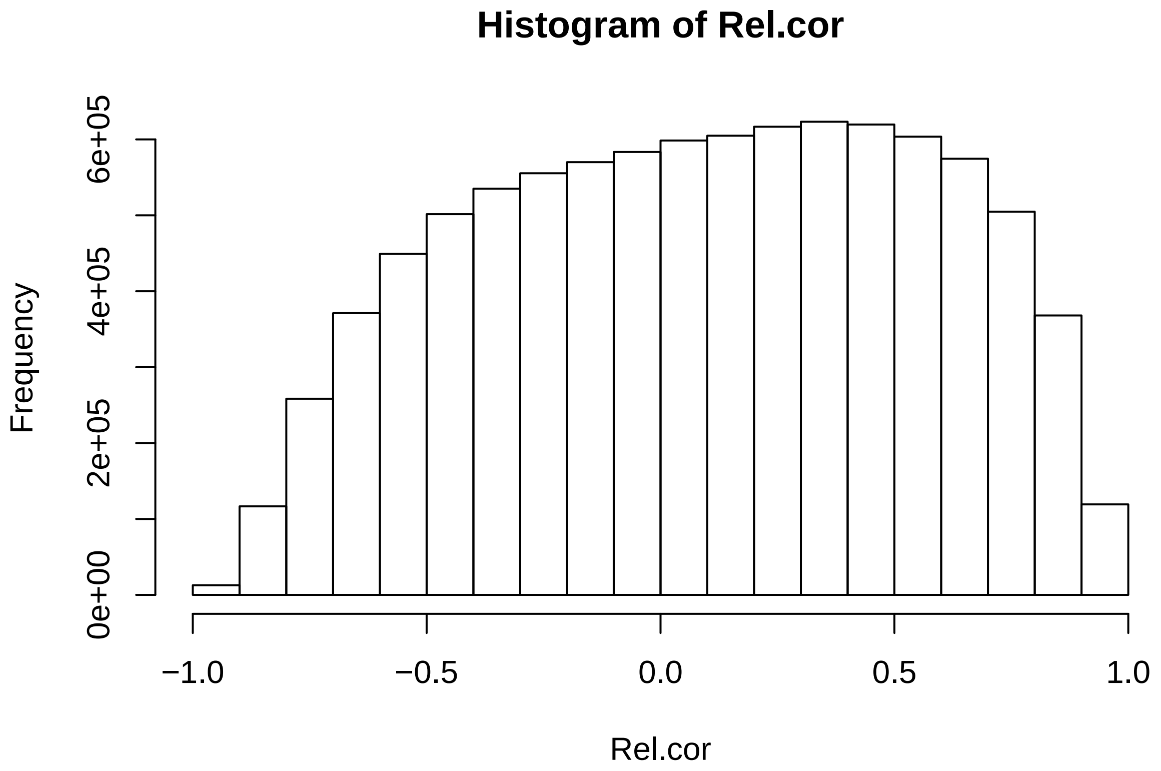

We will not discuss the distribution of these correlations further; rather, we focus now on the agreement between them. For this, we plot the *relative correlations* against the *absolute correlations*. Since correlation matrices have symmetry, we only need to consider one half (e.g., the lower left triangle) of the square matrix. We use the function `propr:::lltRcpp` to pull out this lower left triangle. We sample 5,000 pairs to render the figure quickly.

```
llt <- propr:::lltRcpp
x <- sample(1:length(llt(Abs.cor)), 5000)
plot(llt(Abs.cor)[x], llt(Rel.cor)[x],
     xlab = "Abs", ylab = "Rel", ylim = c(-1,1), xlim = c(-1,1))
```

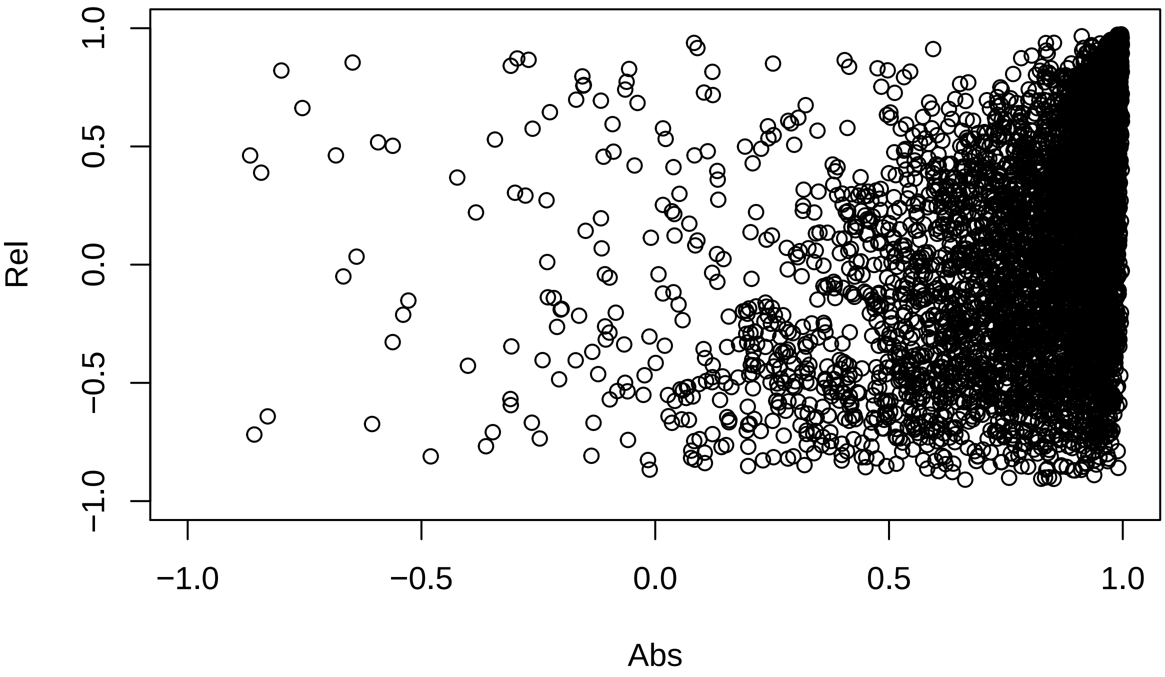

In this figure we see that the relative correlations do not correlate with the absolute correlations. In other words, knowing only the relative correlation tells us nothing about the absolute correlation (Lovell 2015). In fact, some features which appear perfectly correlated in relative space have *negative* correlations in absolute space (i.e., top left of the figure).

When studying a relative dataset, we usually want to know about the true relationships in the corresponding (but unattainable) absolute dataset. In this context, it is maybe helpful to think about relative correlation as a potential proxy for the presence of absolute correlation. As such, we can measure the diagnostic performance of relative correlation as a predictor of absolute correlation. One way to do this is by calculating precision and recall at a number of cutoff values. This results in a contingency table of the agreement between relative and absolute correlation coefficients greater than the cutoff. As an example, we use a cutoff of 0.95 below.

```
table("Observed" = llt(Rel.cor) > .95,
      "Actual" = llt(Abs.cor) > .95)
```

```
##          Actual
## Observed    FALSE    TRUE
##    FALSE 3347355 1233949
##    TRUE       726    9935
```

Here, we define functions for calculating precision and recall from a contingency table like the one above. Note that because each contingency table is specific to the dataset, the precision and recall of relative correlation depends on the dataset too.

```
calculatePrecision <- function(observed, actual){
  conf <- table("Observed" = observed, "Actual" = actual)
  precision <- conf["TRUE", "TRUE"] / (conf["TRUE", "FALSE"] + conf["TRUE", "TRUE"])
}

calculateRecall <- function(observed, actual){
  conf <- table("Observed" = observed, "Actual" = actual)
  recall <- conf["TRUE", "TRUE"] / (conf["FALSE", "TRUE"] + conf["TRUE", "TRUE"])
}
```

We can now calculate the precision and recall of relative correlation across a range of cutoff values. From this, we see that relative correlation is neither a precise nor sensitive indicator of absolute correlation. The preponderance of falsely positive correlations here are known as spurious correlations. The poor agreement between relative correlation and absolute correlation leads us to conclude that correlation is invalid for relative data.

```
result <- NULL
for(cutoff in c(0, .5, .8, .9, .95, .98)){
  p <- calculatePrecision(llt(Rel.cor) > cutoff, llt(Abs.cor) > cutoff)
  r <- calculateRecall(llt(Rel.cor) > cutoff, llt(Abs.cor) > cutoff)
  result <- rbind(result, data.frame(cutoff, p, r))
}
knitr::kable(result)
```

cutoff	p	r
0.00	0.9877582	0.5731141
0.50	0.9681755	0.2524910
0.80	0.9420857	0.0738231
0.90	0.9342019	0.0255395
0.95	0.9319013	0.0079871
0.98	0.9424552	0.0016704

Proportionality is a precise indicator of absolute correlation

Proportionality coefficients, as introduced by Lovell et al. (Lovell 2015) and expounded in Erb & Notredame (Erb 2016), provide a measure of association that is valid for relative data. Although several measures of proportionality exist, we focus here on the metric ρ. This, like correlation, takes on values from $[-1, 1]$, where a value of 1 indicates perfect proportionality. However, unlike correlation, the proportionality coefficient is the same for both relative data and its absolute counter-part, and advantageously tends to produce fewer spurious results. This allows proportionality to serve as a precise indicator of absolute correlation when analyzing relative data.

First, we use the `perb` function from the `propr` package to calculate ρ for all feature pairs. We retrieve the proportionality matrix itself from the `@matrix` slot of the resultant object.

```
library(propr)
rho.clr <- perb(marg.abs)@matrix
```

We can now plot the agreement between ρ and absolute correlation. On inspection, we see immediately that this scatter plot is tapered in such a way that the absolute correlation coefficient is almost always larger than the proportionality coefficient. Since ρ rarely exceeds the absolute correlation coefficient, ρ is rarely falsely positive (i.e., spurious). Moreover, when it is falsely positive, it only inflates the absolute correlation coefficient marginally (unlike relative correlation).

```
plot(llt(Abs.cor)[x], llt(rho.clr)[x],
     xlab = "Abs", ylab = "rho (clr)", ylim = c(-1,1), xlim = c(-1,1))
```

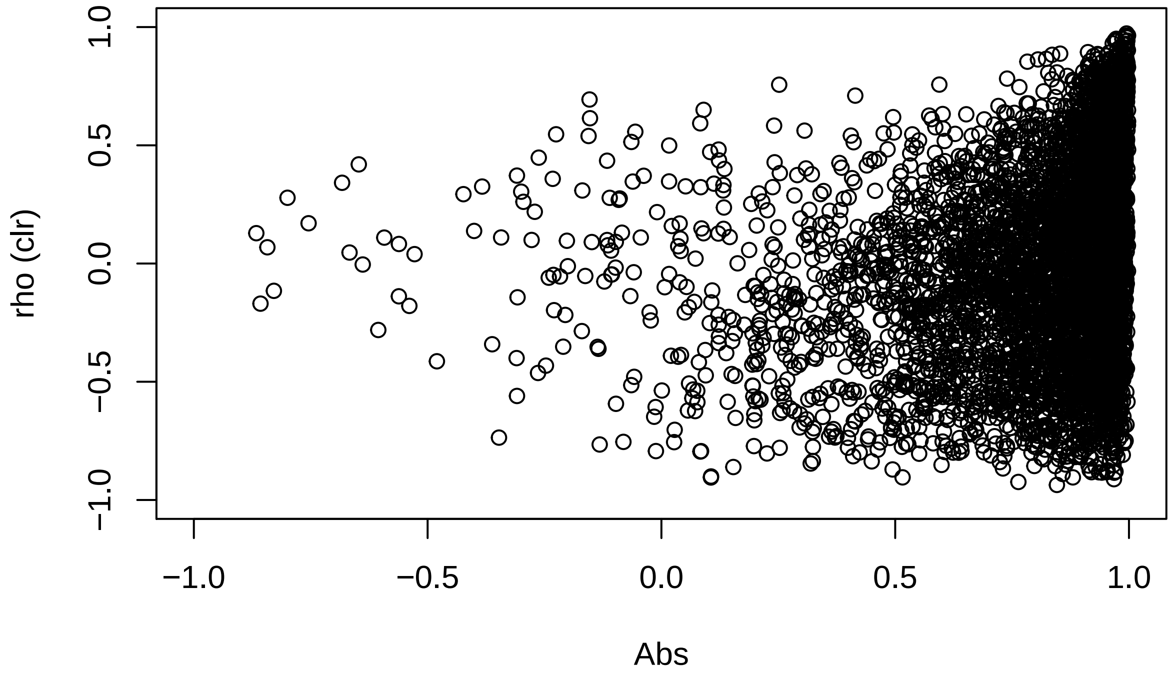

We can now calculate the precision and recall of proportionality across a range of cutoff values. From this, we can see that ρ is a highly precise, albeit not very sensitive, indicator of absolute correlation.

```
result <- NULL
for(cutoff in c(0, .5, .8, .9, .95, .98)){
  p <- calculatePrecision(llt(rho.clr) > cutoff, llt(Abs.cor) > cutoff)
  r <- calculateRecall(llt(rho.clr) > cutoff, llt(Abs.cor) > cutoff)
  result <- rbind(result, data.frame(cutoff, p, r))
}
knitr::kable(result)
```

cutoff	p	r
0.00	0.9834786	0.5013222
0.50	0.9772628	0.1480788
0.80	0.9677951	0.0271903
0.90	0.9595738	0.0069915
0.95	0.9620449	0.0019970
0.98	0.9865471	0.0004986

Why is ρ not sensitive (i.e., has low recall)? Well, part of the definition of proportionality requires calculating the variance of the log-ratio transformed data. One can think of log-ratio transformations as an attempt to scale each subject vector (i.e., time point) by some reference value. This is done by taking the logarithm of each measurement as divided by the reference.

Ideally, this reference is an *unchanged reference*: a feature that has fixed abundance in absolute space across all samples. By default, `propr` uses a *centered log-ratio transformation* in which the geometric mean of the entire subject vector is used as the reference. The better the geometric mean approximates a true unchanged reference, the better ρ agrees with the absolute correlation. The more that ρ agrees with the absolute correlation, the more sensitivity ρ has as an indicator of absolute correlation. Interestingly, proportionality

retains precision even when the geometric mean does not approximate the unchanged reference very well (as in this example).

We support this claim by re-calculating ρ using a different log-ratio transformation. When using the `perb` function, the 'ivar' argument allows us to define a custom reference set to use during the geometric mean calculation. Here, we use the 5 most invariant features (i.e., in absolute space) for the custom reference set. This should provide a reasonable approximation of an idealized unchanged reference. Since we know the absolute abundances, we can determine the invariant features easily. Note that this a contrived example; we most often do not have the absolute abundances when analyzing relative data, or else we would analyze the absolute data directly!

```
i <- order(apply(marg.abs, 2, var))[1:5]
rho.alr <- perb(marg.abs, ivar = i)@matrix
plot(llt(Abs.cor)[x], llt(rho.alr)[x],
     xlab = "Abs", ylab = "rho (alr)", ylim = c(-1,1), xlim = c(-1,1))
```

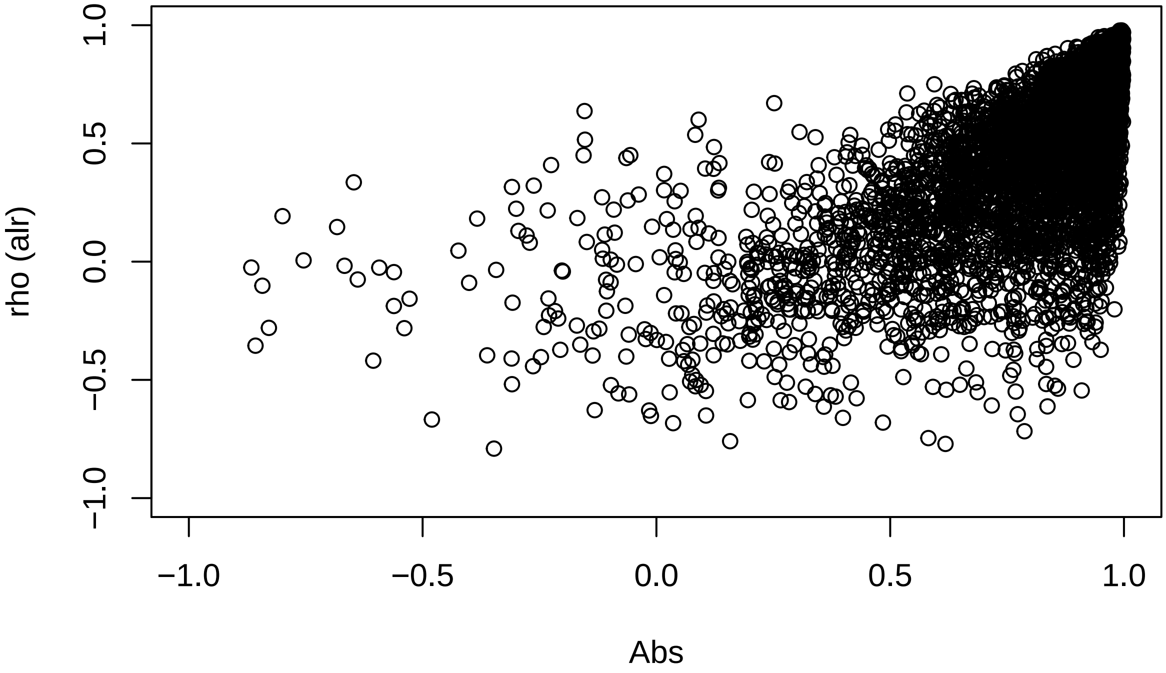

We can now plot the agreement between the reference-based ρ and absolute correlation. Here, we see that using the this invariant reference set improves the agreement between ρ and absolute correlation. This translates to higher recall (and precision too). Importantly, precision remains high regardless of the log-ratio transformation used. The stable precision is what makes proportionality an ideal alternative to correlation in the setting of relative data.

```
result <- NULL
for(cutoff in c(0, .5, .8, .9, .95, .98)){
  p <- calculatePrecision(llt(rho.alr) > cutoff, llt(Abs.cor) > cutoff)
  r <- calculateRecall(llt(rho.alr) > cutoff, llt(Abs.cor) > cutoff)
  result <- rbind(result, data.frame(cutoff, p, r))
}
knitr::kable(result)
```

cutoff	p	r
0.00	0.9938050	0.8991819
0.50	0.9954593	0.5921652
0.80	0.9935975	0.2527057

cutoff	p	r
0.90	0.9907569	0.0977426
0.95	0.9871865	0.0262613
0.98	0.9826938	0.0029601

Selecting a proportionality cutoff based on absolute correlation

Above, we have shown that the proportionality coefficient provides a highly precise estimate for the absolute correlation coefficient. But how proportional is proportional enough?

One way to select a proportionality cutoff is to consider what absolute correlation coefficients we would consider significant. We can determine this easily using Fisher's z'-transformation: this principle states that although the sampling distribution of Pearson's correlation coefficient is not normal, the hyperbolic arctangent transformation of this distribution is normal, and has standard deviation that depends only on the number of samples studied.

$$z = \text{atanh}(r)$$

$$\sigma_z = \frac{1}{\sqrt{N-3}}$$

Since we have established that proportionality is a highly precise (albeit, non-sensitive) indicator of absolute correlation, we could simply choose a statistically sound cutoff for r to stand in place as our proportionalilty cutoff. In other words, we threshold ρ based on a value of r that corresponds to a statistically significant correlation (i.e., for a given sample size and p-value). To do this, we must first determine a cutoff for the transformed value z' based on its the standard deviation. Here, we assume a sample size of 20.

```
N <- 20
sd <- 1/sqrt(N - 3)
```

Knowing standard deviation, we can establish an equality to solve for the z' cutoff. Ultimately, we want to know which value of $\frac{z'}{\sigma_{z'}}$ corresponds to the standard z-score at our desired α cutoff. Here, we choose $\alpha = .05$.

$$z_{\alpha=.05} = \frac{z_{cutoff}}{\sigma_z}$$

Below, we use R to find $z_{\alpha=.05}$ based on a one-tailed test (justified in that we only consider positive correlations here). We then use this value to find the z' cutoff.

```
z05 <- qnorm(.05, lower.tail = FALSE)
z_cutoff <- sd * z05
```

Now, because z' relates to r by the hyperbolic arctangent function, we compute the hyperbolic tangent of z_{cutoff} to find r'_{cutoff}.

```
r_cutoff <- tanh(z_cutoff)
```

Since we have shown that the proportionality coefficient, ρ, provides a highly precise indicator of r, a value of $\rho > r_{cutoff}$ would almost certainly suggest a statistically significant *absolute* correlation coefficient. Although, the converse is again false. Some significant correlations may satisfy $\rho < r_{cutoff}$.

Adjusting the proportionality cutoff for multiple tests

For high-dimensional datasets, we often measure pairwise proportionality between a large number of features. Given D total features, a complete proportionality matrix (i.e., akin to a complete correlation matrix) contains $\frac{D(D-1)}{2}$ unique values. To prevent false discoveries from repeat testing, we must adjust our original α cutoff by the number of tests performed.

As an example, we find the r_{cutoff} for which any correlation coefficient greater would indicate Bonferroni-adjusted significance. As above, we extrapolate this to mean that any $\rho > r_{cutoff}$ would imply (with a high positive predictive value) the presence of a statistically significant *absolute* correlation coefficient.

```
D <- 1000
z_cutoff <- 1 / sqrt(N - 3) * qnorm(.05 / (D * (D - 1)), lower.tail = FALSE)
r_cutoff <- tanh(z_cutoff)
r_cutoff
```

```
## [1] 0.8596171
```

To visualize how r_{cutoff} relates to the sample size, N, and total number of features, D, we build a contour map. This will tell us the correlation coefficient (and, by proxy, the proportionality coefficient) needed to reach Bonferroni-adjusted significance.

```
result <- NULL
for(D in unique(round(2^seq(1, 16, .05)))){
  for(N in unique(4*seq(1, 32))){

    z_cutoff <- 1 / sqrt(N - 3) * qnorm(.05 / (D * (D - 1)), lower.tail = FALSE)
    r_cutoff <- tanh(z_cutoff)
    result <- rbind(result, data.frame(D, N, r_cutoff))
  }
}
knitr::kable(head(result))
```

D	N	r_cutoff
2	4	0.9610871
2	8	0.7046729
2	12	0.5739016
2	16	0.4957053
2	20	0.4425208
2	24	0.4033967

```
library(ggplot2)
library(directlabels)
g <- ggplot(result, aes(x = D, y = N)) + stat_contour(binwidth = .05,
                                                       aes(z = r_cutoff,
                                                           colour = ..level..)) +
  theme_bw() + scale_colour_gradientn(colours = c("black",
                                                  rev(heat.colors(18)),
                                                  "black"),
                                      limits = c(0, 1)) +
  scale_y_continuous(breaks = 4*seq(1, 32)) +
  scale_x_continuous(breaks = 2^(c(0, 12, 14, 15, 16)))
direct.label(g, method = "top.pieces")
```

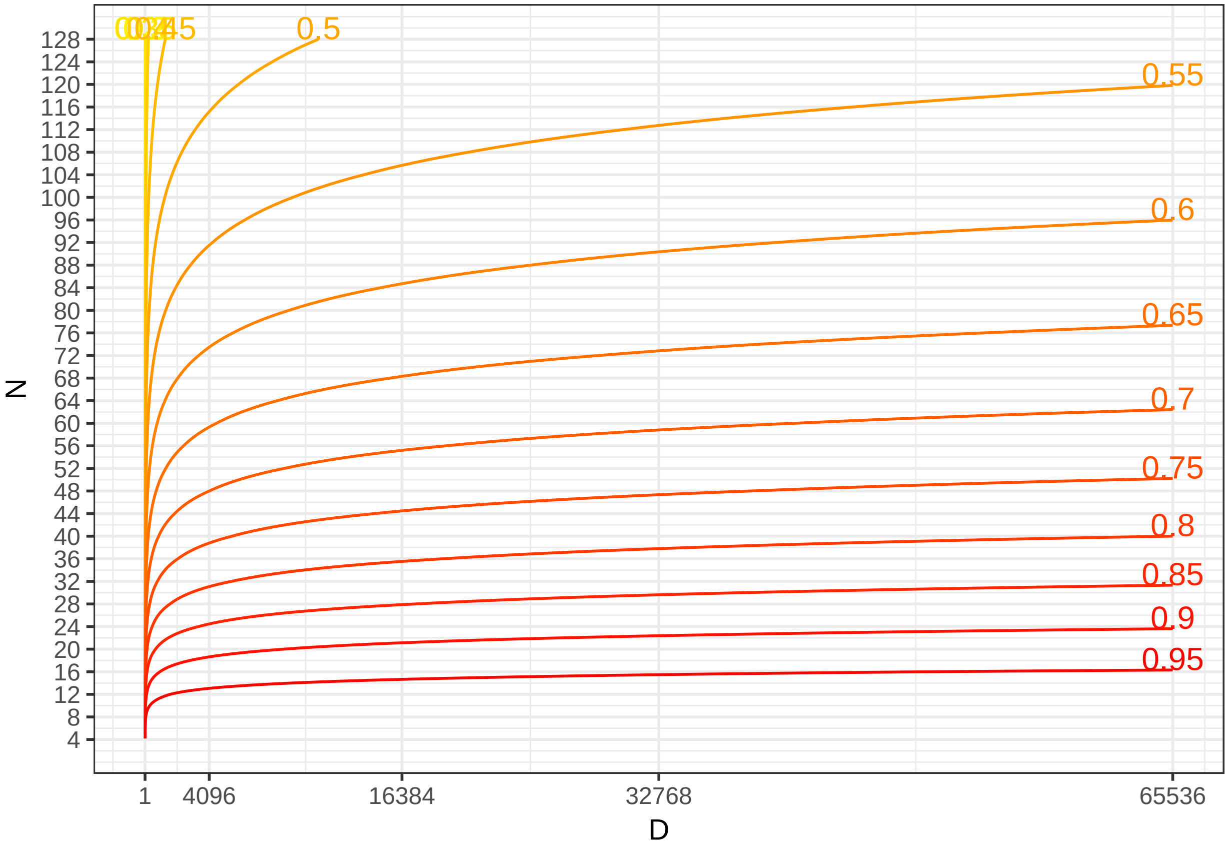

We believe this figure provides a useful "rule of thumb" for selecting a reliable cutoff for the proportionality coefficient, ρ. We acknowledge, however, that using r_{cutoff} might lead to slightly inflated false discovery rates (i.e., above α) due to the fact that ρ does not predict absolute correlation coefficients with exactly 100% precision. We therefore recommend users interpret results cautiously, choosing larger (and therefore more conservative) cutoffs whenever possible.

We also recognize that the ability to calculate an exact p-value for the proportionality coefficients would offer a clear advantage over this method. Extending Lin's formal derivation of the concordance correlation coefficient is one way forward (Lin 1989); an implementation of this is available through the experimental function `propr:::prop2prob`. However, Lin assumes a bivariate normal distribution for the underlying data. There is no evidence that log-ratio transformed data (used in the calculation of ρ) necessarily conform to this distribution. Moreover, uncertainty about the distribution of the underlying *absolute* data presents an additional challenge in calculating exact p-values for ρ.

References

1. Aitchison, J. The Statistical Analysis of Compositional Data. London, UK, UK: Chapman & Hall, Ltd., 1986.

2. Erb, Ionas, and Cedric Notredame. "How Should We Measure Proportionality on Relative Gene Expression Data?" Theory in Biosciences = Theorie in Den Biowissenschaften 135, no. 1–2 (June 2016): 21–36.

3. Lin, L. I. "A Concordance Correlation Coefficient to Evaluate Reproducibility." Biometrics 45, no. 1 (March 1989): 255–68.

4. Lovell, David, Vera Pawlowsky-Glahn, Juan José Egozcue, Samuel Marguerat, and Jürg Bähler. "Proportionality: A Valid Alternative to Correlation for Relative Data." PLoS Computational Biology 11, no. 3 (March 2015): e1004075.

5. Marguerat, Samuel, Alexander Schmidt, Sandra Codlin, Wei Chen, Ruedi Aebersold, and Jürg Bähler. "Quantitative Analysis of Fission Yeast Transcriptomes and Proteomes in Proliferating and Quiescent Cells." Cell 151, no. 3 (October 26, 2012): 671–83.

6. Pearson, Karl. "Mathematical Contributions to the Theory of Evolution. III. Regression, Heredity, and Panmixia." Philosophical Transactions of the Royal Society of London. Series A, Containing Papers of a Mathematical or Physical Character 187 (1896): 253–318.
