# Supplementary material for: propr: An R-package for Identifying Proportionally Abundant Features Using Compositional Data Analysis
Source: Sci Rep. 2017 Nov 24;7:16252. doi: 10.1038/s41598-017-16520-0 (PMC5701231; doi:10.1038/s41598-017-16520-0)
Supplement: Supplementary file 2 — S1 Notes [file 41598_2017_16520_MOESM2_ESM.pdf]

propr: An R-package for Identifying Proportionally Abundant Features Using Compositional Data Analysis

Thomas P. Quinn, Mark F. Richardson, David Lovell, and Tamsyn M. Crowley

```r
###
# Begin proportionality analysis:
#  install.packages("propr")
library(propr)
caneToad.counts <- read.csv("caneToad.csv", row.names = 1)
caneToad.counts <- t(caneToad.counts)
caneToad.groups <- c(rep("WA", 10), rep("QLD", 10))

keep <- apply(caneToad.counts, 2, function(x) sum(x >= 10) >= 10)
rho <- perb(caneToad.counts, select = keep)
best <- rho[">", .95]
best <- simplify(best)

jpeg("fig1.jpeg")
smear(best, prompt = FALSE)
dev.off()

jpeg("fig2.jpeg")
prism(best, k = 5, prompt = FALSE)
dev.off()

clusts <- prism(best, k = 5, prompt = FALSE)
best.1 <- subset(best, select = (clusts == 1))

jpeg("fig3.jpeg")
pca(best.1, group = caneToad.groups, prompt = FALSE)
dev.off()

###
# Integrate with edgeR:
#  source("https://bioconductor.org/biocLite.R")
#  biocLite("edgeR")

library(edgeR)
group <- caneToad.groups
group <- group[group %in% c("WA", "QLD")]
y <- DGEList(counts = t(caneToad.counts), group = group)
y <- calcNormFactors(y)
y <- estimateCommonDisp(y)
y <- estimateTagwiseDisp(y)
et <- exactTest(y)

tt <- as.data.frame(topTags(et, n = nrow(et)))
TTpos <- rownames(tt[tt$FDR < .05 & tt$logFC > 0, ])
TTneg <- rownames(tt[tt$FDR < .05 & tt$logFC < 0, ])
cytescape(best.1[">", .95], prompt = FALSE, col1 = TTpos, col2 = TTneg, d3 = TRUE)

###
# Begin gene ontology (GO) analysis:
#  source("https://bioconductor.org/biocLite.R")
#  biocLite("topGO")
library(topGO)
annots.clean <- yaml::yaml.load_file("caneToadGO.csv")
geneNames <- names(annots.clean)

# For "best.1" subset of propr results
myInterestingGenes <- colnames(best.1@logratio)
geneList <- factor(as.integer(geneNames %in% myInterestingGenes))
names(geneList) <- geneNames
GOdata <- new("topGOdata", ontology = "BP",
              allGenes = geneList, geneSel = myInterestingGenes,
              description = "GO Analysis of a Large Proportional Cluster",
              annot = annFUN.gene2GO, gene2GO = annots.clean)
resultFis <- runTest(GOdata, algorithm = "classic", statistic = "fisher")
allRes <- GenTable(GOdata, classic = resultFis, orderBy = "weight",
                   ranksOf = "classic", topNodes = 20)
write.csv(allRes, "caneToadGO-pr.csv")

# For edgeR results
```

```r
myInterestingGenes <- rownames(tt)[tt$FDR < .05]
geneList <- factor(as.integer(geneNames %in% myInterestingGenes))
names(geneList) <- geneNames
GOdata <- new("topGOdata", ontology = "BP",
              allGenes = geneList, geneSel = myInterestingGenes,
              description = "GO Analysis of a Large Proportional Cluster",
              annot = annFUN.gene2GO, gene2GO = annots.clean)
resultFis <- runTest(GOdata, algorithm = "classic", statistic = "fisher")
allRes <- GenTable(GOdata, classic = resultFis, orderBy = "weight",
                   ranksOf = "classic", topNodes = 20)
write.csv(allRes, "caneToadGO-tt.csv")

###
# Begin propr benchmark:
#  install.packages("magrittr")
library(magrittr)
library(propr)

# Calculate correlation based on the clr-transformation
corr <- function(counts, ivar = 0, select){


  prop <- new("propr", counts = counts, ivar = ivar, select = select)
  prop@matrix <- cor(prop@logratio)
  return(prop)
}

# Compare absolute correlation with relative measures
evaluate <- function(abs, rel){

  cor.abs = propr:::lltRcpp(cor(abs))
  cor.rel = propr:::lltRcpp(cor(rel))
  cor.clr = propr:::lltRcpp(corr(rel)@matrix)
  phi = propr:::lltRcpp(phit(rel)@matrix)
  rho = propr:::lltRcpp(perb(rel)@matrix)
  phs = propr:::lltRcpp(phis(rel)@matrix)

  # Spot check same order
  identical(colnames(phit(rel)@logratio), rownames(cor(abs)))

  df = data.frame(
    cor.abs, cor.rel, cor.clr, rho,
    "phi" = 1-2* plogis(log(phi)),
    "phs" = 1-2 * plogis(log(phs))
  )

  s = sample(1:length(cor.abs), 10000)
  df.sub = df[s, ]

  pairs(
    df.sub, xlim = c(-1, 1), ylim = c(-1, 1),
    labels = c("Correlation\n(Absolute)",
               "Correlation\n(Relative)",
               "Correlation\n(clr-based)",
               "Proportionality\n(perb)",
               "Proportionality,\ntransformed\n(phit)",
               "Proportionality,\ntransformed\n(phis)")
  )

  # Calculate Error for all pairs in lower left triangle
  Eall = data.frame(
    "cor.rel" = (df$cor.abs - df$cor.rel) %>% '^'(2) %>% mean,
    "cor.clr" = (df$cor.abs - df$cor.clr) %>% '^'(2) %>% mean,
    "rho" = (df$cor.abs - df$rho) %>% '^'(2) %>% mean,
    "phi" = (df$cor.abs - df$phi) %>% '^'(2) %>% mean,
    "phs" = (df$cor.abs - df$phs) %>% '^'(2) %>% mean
  )

  # Calculate Error for all pairs *over-estimated*
```

```
  Eover = data.frame(
    "cor.rel" = (df$cor.abs - df$cor.rel)[(df$cor.abs - df$cor.rel) < 0] %>% '^'(2)
%>% mean,
    "cor.clr" = (df$cor.abs - df$cor.clr)[(df$cor.abs - df$cor.clr) < 0] %>% '^'(2)
%>% mean,
    "rho" = (df$cor.abs - df$rho)[(df$cor.abs - df$rho) < 0] %>% '^'(2) %>% mean,
    "phi" = (df$cor.abs - df$phi)[(df$cor.abs - df$phi) < 0] %>% '^'(2) %>% mean,
    "phs" = (df$cor.abs - df$phs)[(df$cor.abs - df$phs) < 0] %>% '^'(2) %>% mean
  )

  # Calculate number of over-estimated pairs
  Eoverfreq = data.frame(
    "cor.rel" = sum((df$cor.abs - df$cor.rel) < 0),
    "cor.clr" = sum((df$cor.abs - df$cor.clr) < 0),
    "rho" = sum((df$cor.abs - df$rho) < 0),
    "phi" = sum((df$cor.abs - df$phi) < 0),
    "phs" = sum((df$cor.abs - df$phs) < 0)
  )

  df = data.frame(
    "E_all" = as.numeric(Eall),
    "E_over" = as.numeric(Eover),
    "Over" = as.numeric(Eoverfreq)
  )

  rownames(df) = colnames(Eall)
  return(df)
}

# Benchmark simulated negative binomial data
# size = mu / 3 := "low variance" scenario in polyester package
set.seed(1)
n = 1000; k = 100
normal <- matrix(runif(n*k), k, n)
nb <- apply(normal, 2, qnbinom, mu = 1000, size = 1000/3)
nb <- cbind(nb, data.frame(n * 500 * (1 + (1:k)/k)))
nb.rel <- t(apply(nb, 1, function(x) x / sum(x)))
jpeg("fig5.jpeg")
out <- evaluate(nb[, -1001], nb.rel[, -1001])
dev.off()
write.csv(out, "out1.csv")

# Benchmark Marguerat data
marg.abs <- read.csv("marguerat.csv", row.names = 1)
marg.rel <- t(apply(marg.abs, 1, function(x) x / sum(x)))
jpeg("fig6.jpeg")
out <- evaluate(marg.abs, marg.rel)
dev.off()
write.csv(out, "out2.csv")
```
